# Identification of Candidate Odorant Receptors in Asian Corn Borer *Ostrinia furnacalis*

**DOI:** 10.1371/journal.pone.0121261

**Published:** 2015-03-24

**Authors:** Bin Yang, Katsuhisa Ozaki, Yukio Ishikawa, Takashi Matsuo

**Affiliations:** 1 Department of Agricultural and Environmental Biology, The University of Tokyo, 1–1–1 Yayoi, Bunkyo-ku, Tokyo, 113–8657, Japan; 2 Biohistory Research Hall, 1–1 Murasaki-cho, Takatsuki, Osaka, 569–1125, Japan; Kansas State University, UNITED STATES

## Abstract

In lepidopteran insects, odorant receptors are involved in the perception of sex pheromones and general odorants. In the Asian corn borer, *Ostrinia furnacalis*, although several pheromone receptors have been identified, no general odorant receptor has been reported. In this study, an RNA sequencing analysis was carried out to identify the whole repertoire of the odorant receptors expressed in the antennae of *O*. *furnacalis*. Among 12 million reads obtained from the antennae of male and female moths, 52 candidate odorant receptors were identified, including 45 novel ones. Expression levels of candidate odorant receptors were estimated by read mapping and quantitative reverse transcription PCR. These analyses confirmed that the expression of the previously identified pheromone receptors was highly male biased. In contrast, none of the newly identified odorant receptors showed male-biased expression. Three of the newly identified odorant receptors showed female-biased expression. Two of them were the most highly expressed odorant receptors in the female antennae, suggesting that they may be involved in the detection of odorants important for the induction of female-specific behaviors such as oviposition site selection. In addition, candidate genes of 21 ionotropic receptors, 5 gustatory receptors, 2 sensory neuron membrane proteins, and 26 odorant degrading enzymes were identified. Our results provide a basis for further analysis of the chemosensory system in the *Ostrinia* species.

## Introduction

Odorant receptors of lepidopteran insects are classified into two major groups, pheromone receptors and general odorant receptors, primarily based on their functions [1, 2]. Pheromone receptors are specialized for the perception of sex pheromones that mediate sexual communication between males and females [3, 4]. Most pheromone receptors are narrowly tuned to the respective components of sex pheromones, and their sensitivity is usually high [5]. On the other hand, general odorant receptors are considered to function in the perception of environmental odorants such as host-plant volatiles, the detection of which is crucial for the selection of oviposition sites [2, 6, 7]. General odorant receptors are as important as pheromone receptors for understanding of the molecular basis of ecological characteristics of each lepidopteran species, but their identification and functional analysis has not been conducted to the same extent compared with those for pheromone receptors [8].

The genus *Ostrinia* (Lepidoptera: Crambidae) comprises 21 species including the Asian corn borer, *Ostrinia furnacalis*, an important agricultural pest [9]. Among the *Ostrinia* species worldwide, the sex pheromones of nine species have been characterized to date [10, 11]. Six pheromone components (Z9–14:OAc, E11–14:OAc, Z11–14:OAc, E12–14:OAc, Z12–14:OAc and E11–14:OH) were identified from these species, and the respective species use different combinations of these components in different proportions for species-specific signaling [12–18]. Nine pheromone receptors (including an odorant receptor coreceptor, *Orco*) have been identified in *O*. *nubilalis*, *O scapulalis*, and *O*. *furnacalis* [12, 19–21]. Electrophysiological analyses by ectopic expression in *Xenopus* oocytes have proven that these receptors in fact respond to the pheromone components [12, 19, 20]. The difference in ligand specificity between orthologous receptors was considered to be involved in the evolution of pheromone communication system in these species [21].

In spite of the intensive analyses of pheromone receptors in *Ostrinia* species, several important issues remain to be addressed. For example, it was not confirmed whether the nine previously identified receptors represent all of the pheromone receptors in the analyzed species. Because these receptors were primarily identified by degenerate PCR based on the conserved sequences at the 5’ and 3’ terminals of ORF sequences [12, 19], it is possible that receptors with divergent structures may have been overlooked. In fact, although the male sex pheromones were shown to be involved in mating acceptance by females in *O*. *nubilalis* [22], most of the previously identified pheromone receptors were reported to be expressed exclusively in the male antennae [12, 19, 20]. Odorant receptors responsible for perception of the male sex pheromones remain to be identified. Besides pheromone receptors, general odorant receptors in *Ostrinia* species are important for understanding of the molecular mechanisms underlying their ecological adaptation, such as host-plant specialization. Considering the fact that many *Ostrinia* species are important agricultural pests, general odorant receptors have the potential to be a target for novel pest control methods. However, no general odorant receptor has been identified in these species.

Besides odorant receptors, many other genes were involved in the odorant perception, such as ionotropic receptors (IRs), gustatory receptors (GRs), sensory neuron membrane proteins (SNMPs), and odorant degrading enzymes (ODEs) [23]. IRs evolved from ionotropic glutamate receptors (iGluRs), and function in detection of acids, amines, and aldehydes [24]. GRs are transmembrane domain receptors mostly expressed in gustatory receptor neurons. However, recent studies suggest that some GRs are expressed in the antennae and involved in the detection of CO_2_ [25]. SNMPs function in the pheromone-detecting ORNs [26]. ODEs are thought to inactivate odorant molecules by enzymatic degradation in the sensillar lymph [23, 27].

In this study, the RNA sequencing (RNA-seq) analysis of *O*. *furnacalis* was conducted to identify the entire repertoire of odorant receptors expressed in the antennae of males and females. We found 45 novel odorant receptor candidates, among which three showed female-biased expression. In addition, candidate genes of 21 IRs, 5 GRs, 2 SNMPs, and 26 ODEs were identified. Our results provide a basis for further study of the molecular mechanisms of chemical perception in the *Ostrinia* species.

## Materials and Methods

### Insect rearing

*O*. *furnacalis* were collected on the Eai river bank (38°35′40″N, 140°57′20″E), Furukawa, Japan, in June 2010. This species is not endangered or protected. Collection of unprotected insects in this area does not require any permission. The collected insects were maintained in the laboratory on the artificial diet for silkworm (Silkmate 2M, Nosan Corporation Life-Tech Department, Yokohama, Japan) at 23°C, under a 16:8 light/dark cycle. The larvae were reared in the insect breeding jar (100 mm diameter × 80 mm height, 310122; SPL Lifesciences Co. Ltd., Seoul, Korea) at a density of 60−80 individuals per bottle until they became pupae. The pupae were collected and divided by sex. Eclosed adults were fed with water for 2 days, then allowed to mate in a net cage containing a plastic cup as the substrate for egg laying.

### RNA sequencing and assembly

Male and female antennae were dissected from 2-day-old adults, and frozen in liquid nitrogen. RNA was immediately isolated from the frozen antennae using the QuickGene RNA tissue Kit SII (RT-s2; KURABO, Neyagawa, Japan). The antennae from more than 20 individuals were pooled for a single RNA isolation experiment. Three biological repeats for each sex were made for the analysis of expression levels. Sequencing libraries were prepared using the TruSeq RNA Sample Preparation Kit v2 according to the LS protocol of the manufacturer’s instructions (Illumina, Inc., San Diego, CA, USA) using 1 μg of total RNA from each sample, except for the following modifications to select the library with long inserts. Incubation time of purified mRNA fragmentation was changed from 8 min to 30 sec at 94°C, and 0.7×volume of the AMPure XP beads was used in the all purification steps. Prepared libraries were mixed at a concentration identical to each other in a 1.5 ml tube and applied for cluster generation on the MiSeq system using the MiSeq Reagent Kit v3 (Illumina, Inc., San Diego, CA, USA). A total of 6 libraries were indexed and applied for a single multiplex run in the 300 bp single-end mode. The raw data were deposited in the DDBJ Sequence Read Archive under accession number DRA002255. The reads were preprocessed with cutadapt v1.2.1 [28] for quality trimming at QV30 with a minimum length of 50 bp. The pass-through reads were pooled and assembled using Trinity r2013_08_14 (http://trinityrnaseq.sourceforge.net/) [29]. Open reading frames were extracted from the Trinity contigs with TransDecoder (http://transdecoder.sourceforge.net/) [30, 31, 32, 33] using the script that came with the Trinity distribution without modification.

### Screening of odorant receptors and read mapping

**Fig 1:**
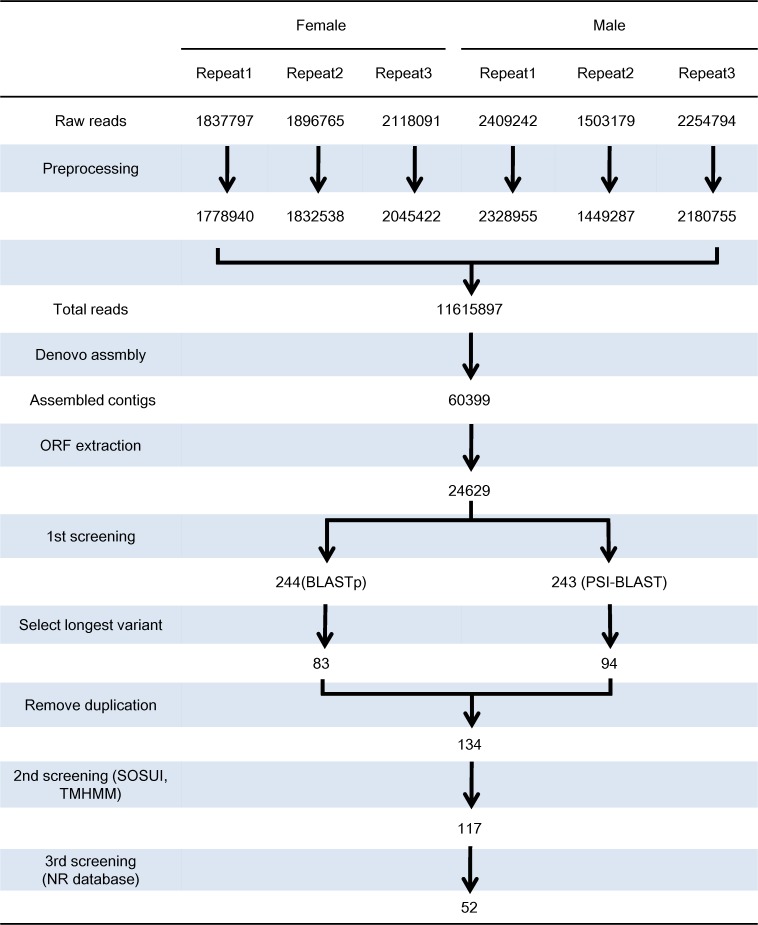
Schematic diagram of sequence data analysis for odorant receptors. The numbers of reads or contigs at each step are indicated. See text for detailed explanation.

The extracted ORF sequences (referred to as the Trinity transcripts hereafter) were first screened by similarity to *Bombyx*. *mori* odorant receptors (*BmorORs*) using two different methods to maximize the possibility of identifying candidate odorant receptors (Fig. 1). A total of 68 protein sequences of *B*. *mori* were obtained from the database [34, 35], and each was used as a query in BLASTp searches against the Trinity transcripts. In parallel, PSI-BLAST searches were performed using alignments of *BmorORs* in various groupings as a query (Table 1). In both searches, the E-value cutoff was set to 0.0001. Overlapping variants were removed at this step by selecting the longest one as a representative transcript of a variant group. The results of two screenings were merged and duplications were removed. The remaining Trinity transcripts were screened for the presence of transmembrane domains using SOSUI (http://harrier.nagahama-i-bio.ac.jp/sosui/) and TMHMM (http://www.cbs.dtu.dk/services/TMHMM/) [36, 37]. The transcripts that contained transmembrane domains were finally screened using BLASTp against the NCBI non-redundant protein database (16.12.2013) (Fig. 1), and those that had an insect odorant receptor as a top-hit homolog were considered as candidate odorant receptors. The expression level of each receptor was estimated by mapping the raw reads to the ORF sequences of the candidate odorant receptors using bowtie2 v2.0.6 in local mode with -a option, followed by processing with eXpress v1.5.1 [38]. Expression levels were calculated as reads per kilobase of the ORF length per million total reads for each library (RPKM) [39].

**Table 1:** Grouping of genes used in the query of the PSI-BLAST search.

Groups	genes from other species
**ORs**	***B*. *mori***
1	*OR26*, *OR45*, *OR46*, *OR47*, *OR48*, *OR50*, *OR51*, *OR57*, *OR58*, *OR59*, *OR63*
2	*Group1*, *OR10*, *OR11*, *OR12*, *OR13*, *OR15*, *OR16*, *OR24*, *OR25*, *OR33*, *OR34*, *OR41*, *OR61*, *OR62*, *OR65*, *OR66*, *OR67*
3	*OR1*, *OR5*, *OR7*, *OR18*, *OR35*, *OR37*, *OR38*, *OR39*, *OR43*, *OR68*
4	*OR5*, *OR7*
5	*OR27*, *OR29*, *OR49*, *OR53*, *OR54*, *OR55*, *OR56*
6	*Group2*, *Group3*, *Group5*
7	*Group6*, *OR3*, *OR23*, *OR28*, *OR42*, *OR64*
8	*Group7*, *OR40*, *OR44*
9	*Group8*, *OR2*, *OR8*, *OR19*, *OR20*, *OR21*, *OR22*, *OR30*, *OR36*
10	*Group9*, *OR4*, *OR6*, *OR9*, *OR14*, *OR17*, *OR32*, *OR52*, *OR60*
**IRs**	***B*. *mori***
1	*IR8a*, *IR25a*, *IR40a*, *IR76b*, *IR93a*
2	*IR7d2*, *IR7d3*, *IR87a*, *IR143*
3	*IR64a*, *IR75d*, *IR75p*, *IR75q2*
4	*Group2*, *IR21a*, *IR41a*, *IR68a*
5	*Group1*, *Group4*
6	*Group3*, *Group5*
**GRs**	***B*. *mori***
1	*GR39*, *GR41*, *GR42*, *GR43*, *GR44*, *GR45*, *GR46*, *GR*, *GR48*, *GR58*, *GR59*, *GR60*, *GR61*, *GR62*
2	*GR12*, *GR13*, *GR24*, *GR25*, *GR26*, *GR27*, *GR28*, *GR29*, *GR30*, *GR31*, *GR32*, *GR33*, *GR34*, *GR35*, *GR36*, *GR37*, *GR38*, *GR40*, *GR47*, *GR64*, *GR65*, *GR68*
3	*GR14*, *GR15*, *GR16*, *GR17*, *GR18*, *GR19*, *GR20*, *GR21*, *GR22*, *GR23*, *GR49*, *GR50*, *GR51*, *GR52*, *GR54*, *GR69*
4	*GR1*, *GR2*, *GR3*, *GR4*, *GR5*, *GR6*, *GR7*, *GR8*, *GR9*, *GR10*, *GR11*, *GR53*, *GR55*, *GR56*, *GR57*, *GR63*, *GR66*, *GR67*
5	*Group2*, *Group3*
6	*Group1*, *Group5*
7	*Group4*, *Group6*
**SNMPs**	***B*. *mori*, *O*. *furnacalis***
1	*BmorSNMP1*,*BmorSNMP2*
2	*OfurSNMP1*, *OfurSNMP2*
3	*BmorSNMP1*, *OfurSNMP1*
4	*BmorSNMP2*, *OfurSNMP2*
5	*BmorSNMP1*,*BmorSNMP2*, *OfurSNMP1*, *OfurSNMP2*
**ODEs**	***S*. *inferens***
1	*CXE2*, *CXE6*, *CXE12*, *CXE14*, *CXE28*
2	*CXE1*, *CXE18*, *CXE20*
3	*Group1*, *Group2*
4	*CXE5*, *CXE9*, *CXE11*, *CXE13*, *CXE16*, *CXE30*
5	*CXE3*, *CXE10*, *CXE26*
6	*Group3*, *Group4*, *Group5*,*CXE19*
7	*AD1*, *AD6*
8	*AD2*, *AD3*, *AD4*, *AD5*
9	*AOX1*, *AOX2*, *AOX3*

### Quantitative reverse transcription PCR

The relative expression levels of the candidate odorant receptors in the antennae and the thorax were validated by quantitative reverse transcription PCR (qRT-PCR). Thorax (mixture from males and females) was used as a control to examine the tissue specificity of the expression pattern. Primers were designed to amplify an approximately 200 bp-long fragment at the 3’ end of the ORF of each candidate (Table 2). Tissues were dissected from 2-days-old adults independently from those used in the RNA-seq analysis. Total RNA was isolated using the QuickGene RNA tissue Kit SII (RT-s2, KURABO, Neyagawa, Japan) and cDNAs were transcribed using the SuperScript III First-Strand Synthesis System (Invitrogen, Carlsbad, CA, USA). Three biological repeats (independent RNA extraction and cDNA synthesis) were made. Quantitative RT-PCR was done using the LightCycler Nano system (Roche, Mannheim, Germany) with the FastStart Essential DNA Green Master Kit (Roche). Three genes, *RpS3*, *actin*, and *NADH dehydrogenase*, were included in the analysis, and the average quantification cycle (Cq) value of these three genes was used as an internal control. Relative expression levels of candidates to the internal controls were estimated as 2^-ΔCq^, where ΔCq represents the difference of Cq between each candidate and the internal control.

**Table 2:** Primers used in qRT-PCR.

Name	Primers
***RpS3***	CAGCTCCCATAGCAATCATGG/CCACGGAAGCATGATCTTTACC
***Actin***	CCGTCCTCCTGACCGAGGCTC/GGTGTGGGAGACACCATCTCCG
***NADH***	GCTGAAGGTGAGAGAGAATTAG/CGAGGTAATGTTCCTCGAACTC
***OfurOR1***	GTGCTGTTCCTGCTCTACAAC/GCTGAACGTTCGCAAGAACATG
***OfurOR2***	GCTCATCAGTGATGGAAGCAG/GCACCAAGTACAGAAGCGAAC
***OfurOR3***	TTGGTACTCAGAGCGAGACCC/GGTGAATGTTCGCAGTAGCATG
***OfurOR4***	GATGTTAGGTGCTGAGACGGAG/TTAATCATTCATTGTTTGTAGG
***OfurOR5a***	GGATTTACAGATGAAGTTTCGGT/GACCGTATATGAGTACAGTCATA
***OfurOR5b***	GGATTTACGGATGAACTTTCGGC/GACCGTATATGAGTAAAGTCAGT
***OfurOR6***	TGCAGTACTACGTTACGGACC/CAGTCCTAATGCCTTGAGACTG
***OfurOR7***	CCTTAGTCTTCGAACTGCTAGG/TAGCAATCATGGTCCTCGAGC
***OfurOR8***	GAGATGTTGGGTTCAGAGACTG/TCTTCAATATCCCGGTCATGG
***OfurOR9***	CAGAGGATGATGGATGCGTGC/TTACGCCATCATTGACCGCAG
***OfurOR10***	CGTACAGTGCCGATTGGATAC/CAGAAGCGTGAAGAACGAGTAC
***OfurOR11***	GGCTTCAATTTATGCCGGTGG/CACTGGTATGATATCAGCAGCC
***OfurOR12***	TTCTGTTGGCACAGCAACGAC/CACTTTGCTGATTCGCAGCTG
***OfurOR13***	ATTGCTGGCACAGCAACGACG/GCCACAGTGAGCTTGGTGAAC
***OfurOR14***	GAGTAGGTGAAGCAGTGTACTG/GAGACGTAGCAAGAGCGTCAATG
***OfurOR15***	GGACTTGTTGAAGAGGAGTCAG/CTCGTGGTTGACATGAACGTG
***OfurOR16***	GAGTGATGGATGCAAGCAAGGC/GCTGAAGCTCAACGTGGTGAC
***OfurOR17***	TAGCTATGGACTGCTGGACTG/CATGAGGCATTCGAAACTCAGC
***OfurOR18***	TTATTATACAGGCGGACCGCG/CACGACAAAAGTGTGGAGATCC
***OfurOR19***	CTCATCGTTTGCTACTGCAGTG/ACCATCGTAAATGTGGCTTGC
***OfurOR20***	GAAAGTACCCTAGTGAGCTACGG/CTGCAGCTTAATCGCAGGATC
***OfurOR21***	ACAGTAGAGAGCGACCGCATG/CAAAGGTGTCAAGTGAGAGCG
***OfurOR22***	GGCACAGTAACGAAGCTTTAG/GAGTGTAGTAGCTGTACGACC
***OfurOR23***	GTGGCCATGCTGCAGATTTAC/GACCACGACGTGCTAATAATC
***OfurOR24***	CATTAGAAGCAGCTCGCATCG/TACGCTGCCTTCATTATCGCG
***OfurOR25***	CTCTTCATGAGCTTGCTGCAAG/GCTCTCAAGTTGACATCTGCG
***OfurOR26***	ATCGCTGCTATGCTACTTCGG/GTAGTGAAGGCCGTCAAGTTC
***OfurOR27***	CGCTAGCAACTATGGAACAGAC/GGTTCCAGCAAGACAATGGTG
***OfurOR28***	CTGAAGTGCGTTTGTGAGAAC/ATCGTGTACATGGAATAAGCC
***OfurOR29***	ACTGCAGTTTATGTGCGCGAC/CACCGAAATGAATGGGCCTGC
***OfurOR30***	GAGTTAACTGCTACTAGCGAAG/ACGAACGTCTGCCTAGACATG
***OfurOR31***	TCGACTGTGAGCAGTCAAGTG/TCAATCTTCTCTTTGGAGCAC
***OfurOR32***	TGGAGCTTAGCTCTATTGAAC/TTACTCTCTCTTGTGCGTTGC
***OfurOR33***	ACAAGTCGATAATGAGTGCGC/ATCCTCCAGAACGGACATGAC
***OfurOR34***	GAGTGGCAGATGCTTTGTATA/CTATGGGTTATAAGTATTGAG
***OfurOR35***	GAACTGATTTGGAAGAGCACTGC/GACTGCGAATGCTTTGTAAGACC
***OfurOR36***	TTGACGTTCGTCGCGAGTATG/GAAGGCCTTCATGACAATCGG
***OfurOR37***	TATGATAGCCGGTTCAGCGTAC/CCTTCCACTTGCTGCAGCAATG
***OfurOR38***	CATCACTATCGAGGCAGCAAG/AACGGAGTATGCTGATTTCACG
***OfurOR39***	AGCGAGAGCGATCAGGTGTGC/GCGAATGTAGTAAGCGAGATGG
***OfurOR40***	CGTATCAGGCTTCACTGTTAC/GAGAATAAGTTCCCTTCAAGAC
***OfurOR41***	AGTTTCACATCTGTCGTCCAC/GAACAGGTTGAAAGCGGTGATG
***OfurOR42***	CATATCGCTAGCAGCATACGAG/TTAATTGACGACGTCTCGGAG
***OfurOR43***	AAATTGCTGACACGATGGCGC/AATGAAGAGCGTAGTTGACGC
***OfurOR44***	ACGATGGTGGCACAGCTGTAC/ACAAAGGTAGGCCTCGACAGG
***OfurOR45***	GATGCGGCATACAATAGTAAATG/CTATTCTGGAGGCTTATAAACCG
***OfurOR46***	GCGTGTTGGGAGATTAGGTTC/GCCTGTCTCAGCATGTTGAAC
***OfurOR47***	CGCTAATACAGCGATGGATGTC/GTCAGCCGAGTTCACGATTTC
***OfurOR48***	GACGGCTTCACCAATATTAAC/TCATTCATCATCATCGTCATC
***OfurOR49***	CACCTTCGACATCCTGTTCATG/TGATGGAGATTGGATGCTGCG
***OfurOR50***	CGATAATCTGCGAATTGCAGCG/GAGTGAAGAATGAGTAGGCCG
***OfurOR51***	TCGGGCTTAGTGTATCATCAGC/GCACTGCATCAGAATCACCATC
***OfurOR52***	GCCTAACCGATGCCATATACTC/GCAGTGCGAAGCAGATTGAAG
***OfurOR53***	GGAGCTATTACCTACGTGAAGC/TTAAGCGCAGGCTGCGTTCATG

### Phylogenetic analysis

Phylogenetic relationships of *O*. *furnacalis* odorant receptors (*OfurORs*) were analyzed against *BmorORs* and *Cydia pomonella* odorant receptors (*CpomORs*) [40, 41]. A total of 164 amino acid sequences were aligned using MAFFT v7.130 with the option E-INS-i [42]. Phylogenetic relationship was deduced by the maximum likelihood method using RAxML v8.0.17 [43, 44] with the GAMMA model for rate heterogeneity and the WAG model for substitution matrix. In addition, the rapid hill-climbing search algorithm (–f d) was used. Model optimization precision in log likelihood units for final optimization of tree topology (–e) was set at 0.0001. The tree image was created using FigTree v1.4.1 (http://tree.bio.ed.ac.uk/software/figtree) [45].

### Identification of other genes involved in the odorant perception

IRs, GRs, SNMPs, and ODEs were identified by the same method used in the identification of odorant receptors, except for the screening by presence of transmembrane domains which was not applied for SNMPs and ODEs. Protein sequences of IRs, GRs, SNMPs in *B*. *mori* were obtained from the database [46, 47, 48, 49]. SNMPs in *O*. *furnacalis* were also used as queries [50]. Because ODEs were not systematically studied in the *B*. *mori*, ODEs in *Sesamia inferens* were used [51].

## Results

### RNA sequencing and screening of candidate odorant receptors

From multiplexed sequencing with Illumina MiSeq for six cDNA libraries, more than 12 million reads were obtained, which consisted of 5,852,653 reads from female antennae and 6,167,215 reads from male antennae (Fig. 1). The average length of reads was approximately 160 bp (Table 3). All reads in a total number of 2 billion bases were pooled together to be assembled into 60,399 contigs, from which 24,629 open reading frame sequences (ORFs) were extracted (Table 4). In the first screening, 244 and 243 sequences were obtained from the homology searches against *B*. *mori* odorant receptors using BLASTp and PSI-BLAST, respectively. These sequences included groups of variants that were identical in their middle section but different from each other in the length of the two termini. Such variants were probably generated by sequencing errors that truncated the deduced ORF. For further analysis, the longest one was selected as a representative sequence of each group. The robustness of this method was confirmed by comparisons with the sequences of previously identified pheromone receptors (see below). After removing duplications between the two screening results (BLASTp and PSI-BLAST), 134 candidates remained. From the second screening using SOSUI and TMHMM, 117 sequences were found to contain transmembrane domains. These sequences were finally screened against the NCBI non-redundant protein database, of which 52 had an insect odorant receptor as the top-hit homolog (Table 5). Seven of the nine previously identified pheromone receptors were found in the candidates, with the exception of *OfurOR1*. Although two sequences were reported for *OfurOR5* (*OfurOR5a* and *OfurOR5b*), only one sequence was found in our candidates, which was slightly different from either. In the other cases, the previously identified receptors and the corresponding candidates were completely identical at the amino acid level but with some differences at the nucleotide level. The 45 newly identified receptors were named from *OfurOR9* to *OfurOR53*.

**Table 3:** Summary of sequencing results.

Reads	Male			Female		
	Repeat1	Repeat2	Repeat3	Repeat1	Repeat2	Repeat3
Total number	1837797	1896765	2118091	2409242	1503179	2254794
Total bases (bp)	300350405	309419147	347769463	390381207	250725980	372529795
Median length (bp)	156	162	160	157	157	158
Q30 percentage	96.80%	96.61%	96.57%	96.67%	96.41%	96.72%

**Table 4:** Summary of assembly results.

	Contigs	ORFs
Total number	60399	24629
Total length (bp)	65431963	29858679
Mean length (bp)	1083	1212
Median length (bp)	617	921
N50 length (bp)	1865	1578

**Table 5:** List of candidate odorant receptors in *O*. *furnacalis*.

Name	Accession Number	aa length	RPKM		RT-PCR**		
			Male	Female	Male	Female	Thorax
*OfurOR1*	AB467327*	425	0.29	0.00	0.000	0.000	0.0000
*OfurOR2*	LC002697	474	678.43	449.04	0.366	0.301	0.0000
*OfurOR3*	LC002698	426	61.07	0.15	0.150	0.000	0.0000
*OfurOR4*	LC002699	423	376.26	0.57	0.383	0.000	0.0000
*OfurOR5a*	AB508302*	408	61.85	1.21	0.199	0.000	0.0000
*OfurOR5b*	AB508303*	408	45.79	1.10	0.234	0.001	0.0000
*OfurOR6*	LC002700	422	37.69	1.01	0.168	0.002	0.0000
*OfurOR7*	LC002701	448	21.43	4.27	0.111	0.014	0.0000
*OfurOR8*	LC002702	438	44.57	0.01	0.162	0.001	0.0000
*OfurOR9*	LC002703	324	27.69	33.48	0.104	0.126	0.0001
*OfurOR10*	LC002704	404	17.35	31.60	0.046	0.066	0.0000
*OfurOR11*	LC002705	398	15.75	16.22	0.027	0.021	0.0000
*OfurOR12*	LC002706	407	13.95	16.74	0.036	0.027	0.0000
*OfurOR13*	LC002707	425	12.75	19.00	0.022	0.019	0.0000
*OfurOR14*	LC002708	424	12.32	23.11	0.027	0.038	0.0000
*OfurOR15*	LC002709	423	12.11	88.09	0.034	0.210	0.0000
*OfurOR16*	LC002710	422	10.16	22.84	0.019	0.023	0.0000
*OfurOR17*	LC002711	239	10.08	6.46	0.034	0.043	0.0000
*OfurOR18*	LC002712	471	9.72	14.54	0.012	0.012	0.0001
*OfurOR19*	LC002713	422	8.93	15.96	0.039	0.047	0.0000
*OfurOR20*	LC002714	411	8.79	12.19	0.018	0.018	0.0000
*OfurOR21*	LC002715	363	8.37	8.91	0.003	0.003	0.0000
*OfurOR22*	LC002716	409	8.27	14.28	0.022	0.028	0.0000
*OfurOR23*	LC002717	404	7.71	14.27	0.023	0.039	0.0000
*OfurOR24*	LC002718	448	6.44	8.52	0.014	0.017	0.0005
*OfurOR25*	LC002719	418	6.14	8.19	0.004	0.002	0.0000
*OfurOR26*	LC002720	433	6.01	7.07	0.011	0.015	0.0000
*OfurOR27*	LC002721	402	5.94	6.89	0.012	0.020	0.0001
*OfurOR28*	LC002722	420	5.93	11.78	0.022	0.022	0.0001
*OfurOR29*	LC002723	441	5.71	12.74	0.008	0.014	0.0000
*OfurOR30*	LC002724	402	5.58	10.89	0.010	0.014	0.0000
*OfurOR31*	LC002725	396	5.43	6.52	0.008	0.009	0.0001
*OfurOR32*	LC002726	412	5.11	14.27	0.004	0.006	0.0000
*OfurOR33*	LC002727	410	5.06	7.58	0.023	0.028	0.0001
*OfurOR34*	LC002728	439	5.01	8.82	0.008	0.008	0.0000
*OfurOR35*	LC002729	430	4.76	7.34	0.009	0.011	0.0003
*OfurOR36*	LC002730	398	4.72	5.60	0.001	0.001	0.0000
*OfurOR37*	LC002731	390	4.69	5.36	0.008	0.008	0.0000
*OfurOR38*	LC002732	415	4.59	7.55	0.010	0.009	0.0001
*OfurOR39*	LC002733	386	4.46	44.89	0.010	0.064	0.0000
*OfurOR40*	LC002734	432	4.44	10.99	0.005	0.006	0.0000
*OfurOR41*	LC002735	421	4.24	13.06	0.008	0.025	0.0004
*OfurOR42*	LC002736	346	4.16	7.13	0.010	0.015	0.0000
*OfurOR43*	LC002737	198	4.00	8.34	0.008	0.010	0.0003
*OfurOR44*	LC002738	436	3.54	4.33	0.003	0.006	0.0000
*OfurOR45*	LC002739	407	3.51	6.69	0.013	0.013	0.0000
*OfurOR46*	LC002740	431	3.05	8.02	0.015	0.016	0.0006
*OfurOR47*	LC002741	103	2.86	1.63	0.002	0.003	0.0000
*OfurOR48*	LC002742	265	2.65	7.15	0.037	0.015	0.0001
*OfurOR49*	LC002743	361	2.49	5.72	0.006	0.010	0.0002
*OfurOR50*	LC002744	355	1.88	4.52	0.003	0.006	0.0000
*OfurOR51*	LC002745	380	1.56	2.86	0.006	0.008	0.0000
*OfurOR52*	LC002746	409	0.79	6.31	0.002	0.003	0.0000
*OfurOR53*	LC002747	407	0.00	7.55	0.000	0.018	0.0000

*: Reported in the previous paper [12].

**: Relative expression level to the internal control.

### Expression levels of the candidate odorant receptors estimated by read mapping

**Fig 2:**
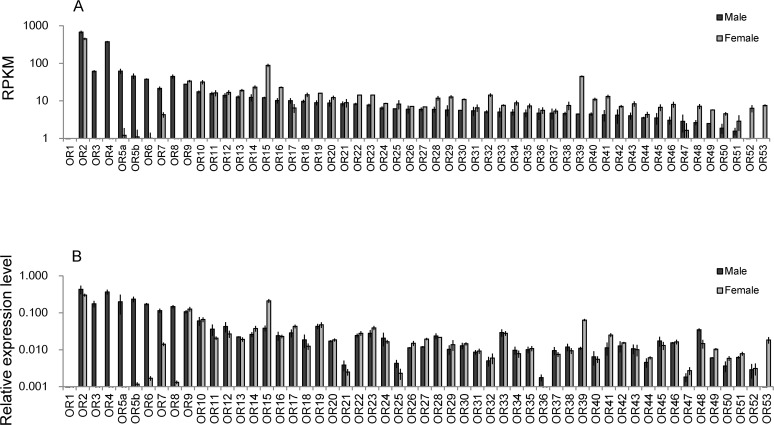
Expression level of candidate odorant receptors in adult antennae. (A) Estimated by read mapping. (B) Estimated by qRT-PCR. Error bars represent standard error calculated from the results of three biological replicates.

To estimate the expression level of the candidate odorant receptors in males and females, the reads were mapped onto the ORF sequences of the candidate receptors. Because *OfurOR1* was not found in our candidates, its sequence was obtained from the database. Sequences for *OfurOR5a* and *OfurOR5b* were also obtained from the database and treated as independent receptors in the mapping. As expected, *OfurOR2* (*Orco*) was expressed at the highest level in both male and female antennae (Table 5, Fig. 2A). Most of the previously identified pheromone receptors (*OfurOR3*, *4*, *5a*, *5b*, *6*, *7*, and *8*) showed male-specific expression, which was consistent with previous studies [12, 19, 20]. Among these, *OfurOR4*, the receptor for the major pheromone component in *O*. *furnacalis*, was expressed at the highest level. *OfurOR7* was expressed not only in males but also in females at an intermediate level. Surprisingly, but consistently with the results of the candidate screening, the number of reads mapped onto *OfurOR1* was very low, suggesting that it was not expressed in our samples. None of the 45 novel candidate receptors showed strongly male-biased expression as observed with the previously identified pheromone receptors. Because the read counts were normalized by the total read number, and a large part of the reads were mapped onto the pheromone receptors in males, the RPKM values for the other receptors tended to be higher in females. Nevertheless, *OfurOR15*, *39*, *52*, and *53* should be recognized as female-biased receptors. In particular, *OfurOR15* and *OfurOR39* were expressed at the next highest levels after *OfurOR2* (*Orco*) in female antennae.

### Expression levels of the candidate odorant receptors confirmed by qRT-PCR

To confirm the expression levels of the candidate odorant receptors, qRT-PCR was carried out using the independently prepared cDNA libraries. The primers for qRT-PCR were designed to specifically recognize the sequence at the 3’ end of each candidate (Table 2). Thorax cDNA libraries were used as the negative control, and no expression was detected for any of the odorant receptors (Table 5). In the antennae, the results were generally consistent with those of the read mapping but with some exceptions (Fig. 2B). The inconsistency with the results of read mapping was probably caused by high sequence similarity between two receptors. Because we used -a option in the bowtie2 mapping, single reads derived from the high-homology regions were mapped to both of the receptors with a 0.5 count each, resulting in a similar RPKM value in both receptors. Such cases were likely in *OfurOR21*, *25*, and *36* that were expressed at lower levels than estimated by the read mapping. Female-biased expression was confirmed for *OfurOR15*, *39*, and *53*. In particular, *OfurOR53* was highly female-specific, suggesting its dedicated role in females. Expression of *OfurOR7* in females was also confirmed. Because the male-female expression ratio was more accurately estimated by qRT-PCR than read mapping, the difference between males and females was smaller for most receptors than that estimated by read mapping.

### Phylogenetic analysis

**Fig 3:**
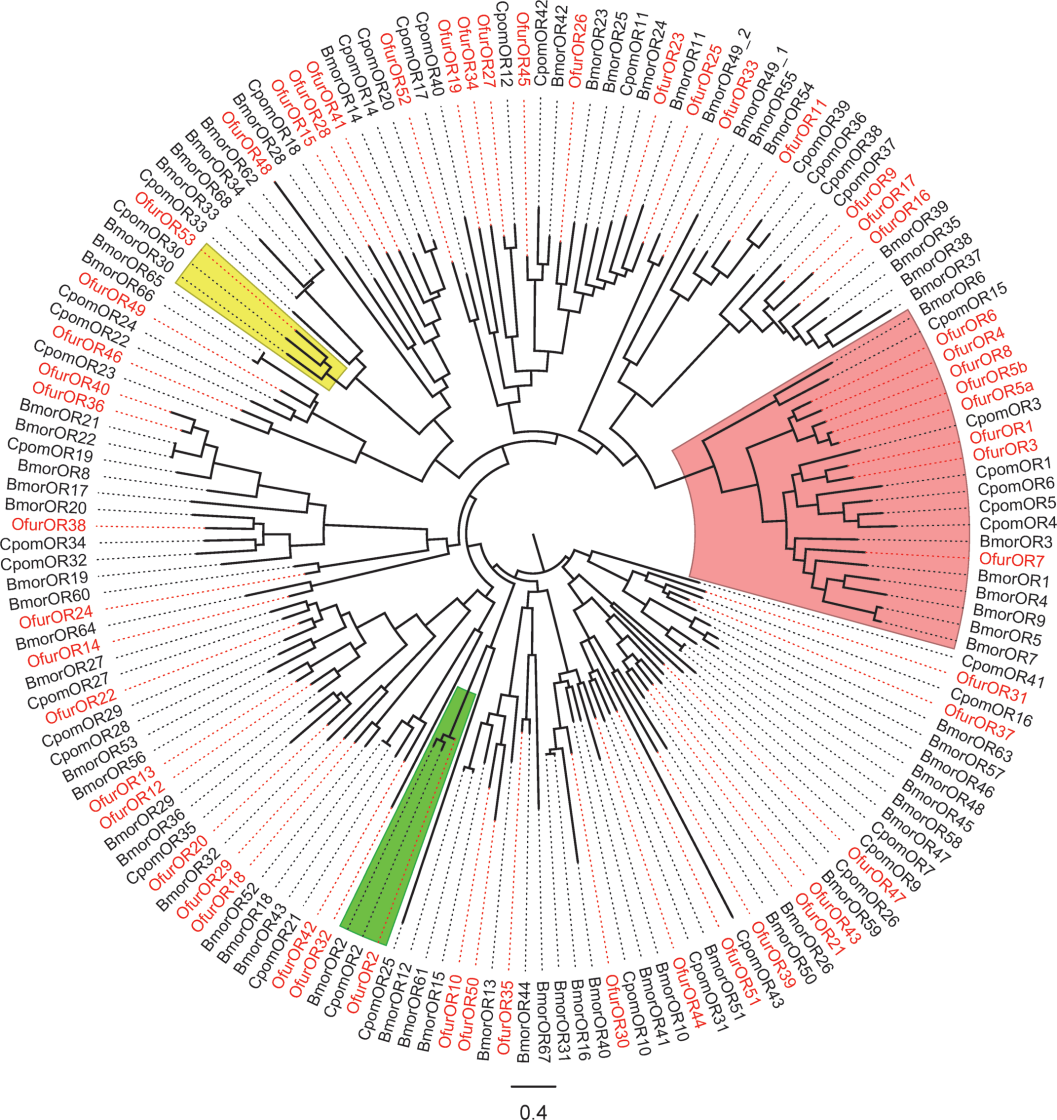
Phylogenetic relationship of *O. furnacalis* odorant receptors (*OfurORs*) with those of B. mori (*BmorORs*) and C. pomonella (*CpomORs*). The tree was constructed by the maximum likelihood method using RAxML and visualized using FigTree. *OfurORs* are indicated in red. Green, pink, and yellow shading indicates the clades of *Orco*, pheromone receptors, and *OfurOR53*, respectively.

Phylogenetic relationships between *OfurORs* and *BmorORs*, as well as with odorant receptors in *C*. *pomonella* are shown in Fig. 3. As expected, *Orco* was highly conserved among the three species. All the previously identified pheromone receptors of *O*. *furnacalis* formed a single clade with other pheromone receptors from *B*. *mori* and *C*. *pomonella*. Within this clade, however, receptors from the same species tended to form subclusters, suggesting that pheromone receptors have undergone species-specific duplication events. The female-specific receptor *OfurOR53* formed a clade with *BmorOR30* and *CpomOR30*, among which *BmorOR30* was reported to exhibit female-specific expression [34, 40], whereas *CpomOR30* was not [41]. The other two female-biased receptors, *OfurOR15* and *OfurOR39*, belonged to independent clades. *OfurOR15* formed a clade with *OfurOR28*, *OfurOR41*, *BmorOR14*, *CpomOR14*, and *CpomOR20*. Among these, *OfurOR41* showed slightly female-biased expression (Fig. 2B), but the others were expressed both in males and females [34, 40, 41]. *OfurOR39* formed a clade with *OfurOR51*, *BmorOR50*, *BmorOR51*, and *CpomOR43*. None of these were reported to be female biased [34, 40, 41].

### Identification of other genes involved in the odorant perception

**Fig 4:**
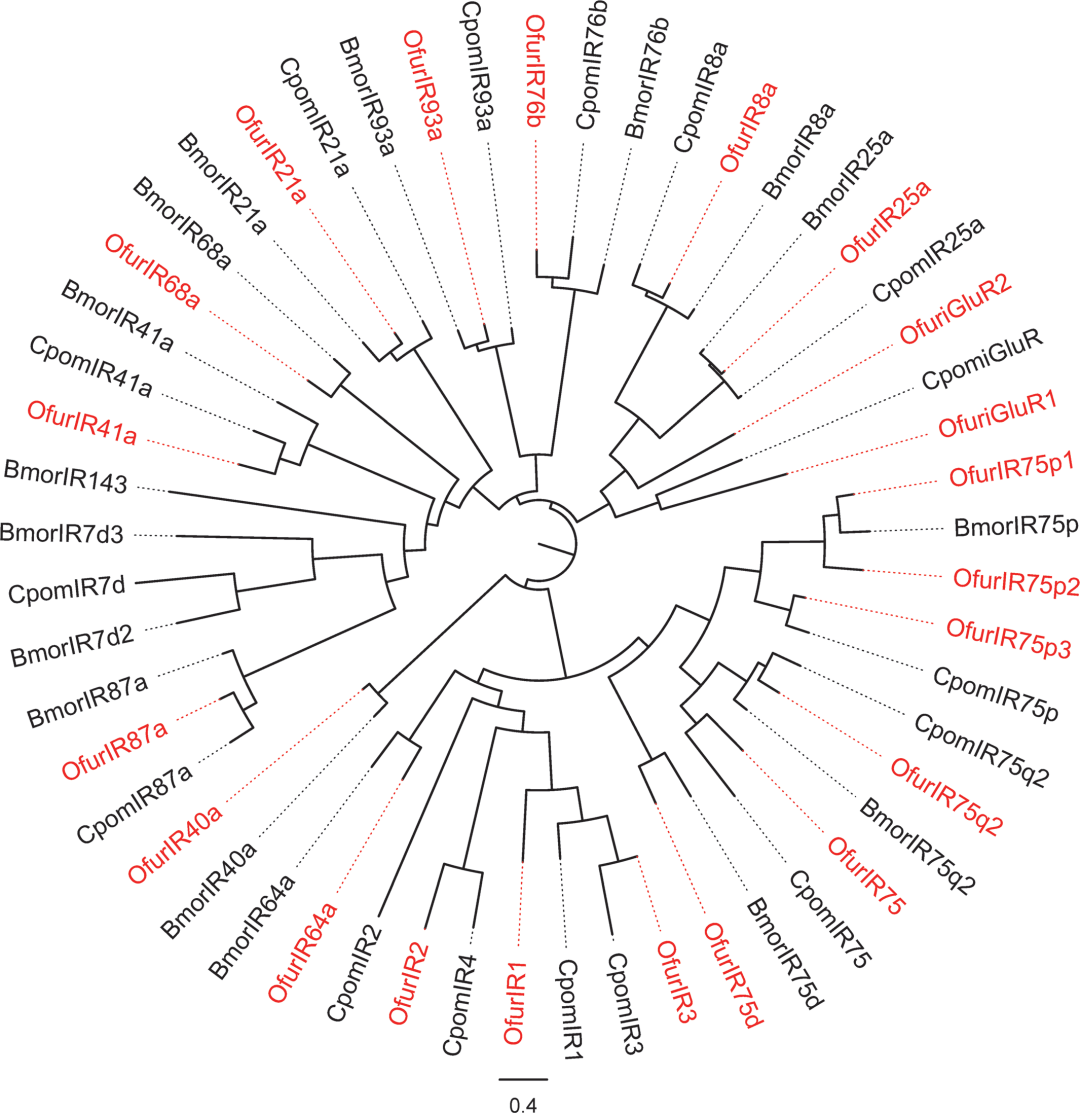
Phylogenetic relationship of *O. furnacalis* ionotropic receptors (*OfurIRs*) with those of B. mori (*BmorIRs*) and C. pomonella (*CpomIRs*). The tree was constructed by the maximum likelihood method using RAxML and visualized using FigTree. *OfurORs* are indicated in red.

We also identified candidate genes of 21 IRs, 5 GRs, 2 SNMPs and 26 ODEs (Table 6). All the genes were novel in *O*. *furnacalis* except for SNMPs [50]. The phylogenetic relationships between *OfurIRs*, *BmorIRs*, and *CpomIRs* are shown in Fig. 4. ODEs were divided into three families, including eight aldehyde oxidases (*OfurAOX1* to *OfurAOX8*), fifteen carboxylesterase (*OfurCXE1* to *OfurCXE15*) and three alcohol dehydrogenase (*OfurAD1* to *OfurAD3*) (Table 6). Most of the identified genes were full length. However, all of the GR genes were partial, probably due to their low expression levels in the antennae.

**Table 6:** List of other candidate genes involved in olfactory perception in *O*. *furnacalis*.

Name	Accession Number	aa length	RPKM	
			Male	Female
*OfuriGluR1*	LC017780	923	5.67	7.99
*OfuriGluR2*	LC017781	900	5.93	6.37
*OfurIR8a*	LC017782	902	37.14	50.82
*OfurIR21a*	LC017783	849	26.88	36.66
*OfurIR25a*	LC017784	942	71.03	68.03
*OfurIR40a*	LC017785	709	3.26	5.41
*OfurIR41a*	LC017786	596	12.15	17.01
*OfurIR64a*	LC017787	606	5.79	8.17
*OfurIR68a*	LC017788	340	4.34	6.67
*OfurIR75*	LC017789	626	13.28	16.00
*OfurIR75d*	LC017790	274	2.29	3.13
*OfurIR75p1*	LC017791	630	4.40	10.33
*OfurIR75p2*	LC017792	609	5.42	10.47
*OfurIR75p3*	LC017793	639	3.30	0.00
*OfurIR75q2*	LC017794	637	6.99	11.58
*OfurIR76b*	LC017795	547	27.01	46.73
*OfurIR87a*	LC017796	654	5.25	7.12
*OfurIR93a*	LC017797	890	6.64	7.06
*OfurIR1*	LC017798	358	2.02	4.12
*OfurIR2*	LC017799	357	9.06	18.22
*OfurIR3*	LC017800	178	1.11	1.65
*OfurGR1*	LC017775	140	1.62	3.25
*OfurGR2*	LC017776	130	2.36	0.00
*OfurGR3*	LC017777	121	1.05	1.47
*OfurGR4*	LC017778	100	0.00	2.50
*OfurGR5*	LC017779	194	2.36	3.38
*OfurSNMP1*	LC017801	528	919.31	389.54
*OfurSNMP2*	LC017802	523	1352.03	1317.27
*OfurAOX1*	LC017752	1275	134.36	161.16
*OfurAOX2*	LC017753	1279	189.54	297.30
*OfurAOX3*	LC017754	1280	19.36	15.92
*OfurAOX4*	LC017755	766	6.35	10.49
*OfurAOX5*	LC017756	593	3.36	6.88
*OfurAOX6*	LC017757	1268	13.28	13.98
*OfurAOX7*	LC017758	778	7.68	5.12
*OfurAOX8*	LC017759	378	2.44	4.03
*OfurCXE1*	LC017760	560	5.93	5.25
*OfurCXE2*	LC017761	541	135.42	116.35
*OfurCXE3*	LC017762	532	11.35	14.77
*OfurCXE4*	LC017763	559	66.51	34.96
*OfurCXE5*	LC017764	566	15.20	19.43
*OfurCXE6*	LC017765	511	13.59	15.40
*OfurCXE7*	LC017766	317	82.91	68.76
*OfurCXE8*	LC017767	566	354.93	237.89
*OfurCXE9*	LC017768	544	23.12	30.35
*OfurCXE10*	LC017769	542	23.89	28.67
*OfurCXE11*	LC017770	527	22.83	32.79
*OfurCXE12*	LC017771	519	17.16	30.97
*OfurCXE13*	LC017772	511	44.64	61.26
*OfurCXE14*	LC017773	562	175.11	229.03
*OfurCXE15*	LC017774	515	2.32	5.19
*OfurAD1*	LC017749	325	32.41	43.19
*OfurAD2*	LC017750	356	5.34	8.79
*OfurAD3*	LC017751	365	46.62	53.06

## Discussion

### Pheromone receptors

In the previous study, pheromone receptors in *O*. *furnacalis* were cloned by degenerate PCR [12]. For this reason, the 5’ and 3’ terminal sequences of the ORFs were not known. In the present study, we identified complete ORF sequences for seven of the nine previously identified pheromone receptors. On the other hand, *OfurOR1* was not found in our RNA-seq analysis. It was also not detected in the independent qRT-PCR analysis, indicating that *OfurOR1* was not expressed in our sample. This might be due to intraspecies polymorphism because our samples and those used in the previous studies were derived from different localities in Japan [12]. None of the 45 novel receptors found in this study showed male-biased expression as observed in the previously identified pheromone receptors. The previously identified pheromone receptors were structurally distinct from the other receptors; they formed a single clade in the phylogenetic analysis. Thus, it is likely that there are no additional pheromone receptors in *O*. *furnacalis* other than the already identified ones. However, the presence of some other receptors that incidentally respond to pheromone components was not excluded. Identification of novel odorant receptors in *O*. *furnacalis* provides the opportunity to experimentally examine this possibility.

### Phylogenetic relationship of odorant receptors with sexually biased expression

Genome wide analysis of the expression pattern of odorant receptors has been carried out in several lepidopteran species including *B*. *mori* [34, 40], *Manduca sexta* [52], *C*. *pomonella* [41], *Helicoverpa armigera* [53], and *Spodoptera littoralis* [54]. In each species, receptors with sex-specific expression have been identified. Some of these receptors are phylogenetically close to each other. The most significant example is the pheromone receptor group, which contains nine receptors from three species (*BmorOR1*, *3*, *4*, *5*, *6*; *HarmOR14*, *15*; *SlitOR6*, *13*) that were male specific [34, 53, 54]. The previously identified pheromone receptors in *O*. *furnacalis* belonged to this group, and most of them were male specific [12]. However, not all of the members were male biased. Seven receptors from three species (*CpomOR3*, *5*; *HarmOR1*, *2*, *11*; *SlitOR11*, *16*) were equally expressed in males and females [41, 53, 54]. Furthermore, *CpomOR15* was shown to be female specific [41]. Another example is a group of receptors including *OfurOR53* and *BmOR30*. Although these two receptors were specifically expressed in female antennae ([34, 40], this study), orthologous receptors in other species (*CpomOR30* and *SlitOR30*) were also expressed in the male antennae [41, 54]. These examples indicated that sexually biased expression is under the influence of phylogenetic constraint to some extent, but it also evolves dynamically from sex-specific expression to sex-independent expression and vice versa. Nevertheless, it should be noted that in most of the previous studies, the expression levels were determined by non-quantitative methods, leaving the possibility that the difference between sexes was over- or under-estimated [34, 41, 52–54]. Quantitative analysis of the expression level is necessary to gain insight into the evolutionary pattern of sexually biased expression of odorant receptors.

### Biological function of female-biased receptors in *O*. *furnacalis*

In this study, the expression levels of the all receptors were estimated quantitatively by two independent methods, which demonstrated that *OfurOR53*, *15*, and *39* had female-biased expression. Importantly, the latter two were the receptors with the highest expression level in female antennae next to *OfurOR2* (*Orco*). One possible function of these receptors is the perception of male sex pheromone, which was reported to be required for acceptance of mating by females in *O*. *nubilalis* [21]. The *OfurOR7* is also a candidate for the male pheromone receptor. It belongs to the pheromone receptor group, and it was also expressed in the female antennae. Another possible function of the female-biased receptors is to recognize host-plant volatiles. Finding an appropriate host plant is crucial for reproduction in the herbivorous lepidopteran insects. Odorant receptors involved in host-plant detection would serve as a potential target for novel pest control techniques. In this regard, *OfurOR15* and *OfurOR39*, the receptors with the highest expression levels in female antennae, should be considered as the primary candidates for further characterization of their molecular function.

### Repertoire of odorant receptors in *O*. *furnacalis*

Although an intensive analysis of the antennal transcriptome was conducted in this study, other tissues were not investigated. Therefore, odorant receptors not expressed in the antennae were not included in our analysis. Furthermore, receptors with extremely low expression levels may not have been identified. In fact, the ORF sequences appeared to be incomplete for a few receptors with low expression levels (Table 5). Two receptors with a similar sequence, such as recently duplicated pairs, were indistinguishable in our analysis, as seen in the case of *OfurOR5a* and *OfurOR5b*. Finally, extremely divergent receptors that were not similar to any of the other insect odorant receptors may not be identified in our analysis, although the candidates excluded at the third screening (homology search against the NCBI nr database) were significantly similar to non-odorant-receptor proteins. These limitations mean that our method is conservative, and whole genome sequence analysis may identify additional odorant receptors in *O*. *furnacalis*. Nevertheless, our results provide a list of odorant receptors with significant expression in the antennae, thus they are considered to be biologically functional. Our present results will serve as a basis for studies to understand the evolution of the pheromone communication system, as well as for the development of novel control methods of agriculturally important pests.
